# The Relationship between Gelation Behavior and the Amount of Polymer Dose per Silica Surface Area of “Shake-Gels” Consisting of Silica Nanoparticles and Poly(Ethylene Oxide)

**DOI:** 10.3390/molecules28083555

**Published:** 2023-04-18

**Authors:** Shunsuke Sato, Motoyoshi Kobayashi

**Affiliations:** 1 Graduate School of Science and Technology, University of Tsukuba, 1-1-1 Tennoudai, Tsukuba 305-8572, Japan; 2 Faculty of Life and Environmental Sciences, University of Tsukuba, 1-1-1 Tennoudai, Tsukuba 305-8572, Japan

**Keywords:** shear-induced gels, rheology, gelation time, flow type, poly(ethylene glycol)

## Abstract

The understanding and control of the rheological behaviors of colloids and polymer mixtures is an important issue for scientific interests and industrial applications. Aqueous mixed suspensions of silica nanoparticles and poly(ethylene oxide) (PEO) under certain conditions are interesting systems called “shake-gels”, whose states vary reversibly between sol-like and gel-like under repeated shaking and being left to stand. Previous studies have indicated that the amount of PEO dose per silica surface area (*C*_p_) is a crucial parameter for the formation of shake-gels and the relaxation time from gel-like to sol-like states. However, the relationship between the gelation dynamics and the *C*_p_ values has not been fully investigated. To determine how the gelation dynamics are affected by the *C*_p_, we measured the time taken for silica and PEO mixtures to gelate from the sol-like to gel-like states as a function of the *C*_p_ under different shear rates and flow types. Our results show that the gelation time decreased with increasing shear rates and depended on the *C*_p_ values. Moreover, the minimum gelation time was found around a certain *C*_p_ (=0.03 mg/m^2^) for the first time. The finding suggests that there is an optimum *C*_p_ value at which the bridging of silica nanoparticles using PEO is significant, and thus, the shake-gels and stable gel-like states are most likely to form.

## 1. Introduction

Colloidal suspensions appear in a variety of situations in engineering and natural processes, such as drilling fluids [1], coating of functional materials [2], adsorption or transportation of pollutants [3], and so on. Understanding and controlling the rheological behaviors of the colloidal suspensions is useful for applications or further understanding in these fields [1,2,3,4,5]. Colloidal suspensions generally exhibit non-Newtonian flow behaviors in which the viscosity changes with shear rate due to repulsive double-layer interaction and formation/breakup of colloidal aggregates [2,3,4,5]. In particular, shear thickening and rheopexy, which are the increase in viscosity with shear rate and time, can be problems for many industrial processes because they lead to energy loss, machine failure, and lower product quality [2,5]. Conversely, the thickening phenomenon has been proposed to be applied to shock-absorbing materials [6]. As a consequence, elucidating the mechanism of the thickening phenomenon and its control leads to preventing problems in industrial processes and the creation of new materials.

Adding polymers to suspensions is a widely used technique for controlling the aggregation/dispersion behavior and rheological properties of suspensions. The added polymers cause steric repulsion, bridging flocculation, and depletion attraction between the particles [7,8,9]. The particle-polymer interaction is affected by various parameters such as particle size, polymer branching structure, temperature, pH, the existence of flow, and so on [10,11,12]. In particular, the coverage of polymer on the particle surfaces is an important parameter for the action of adsorbed polymers since the low coverage causes flocculation due to the bridging, and sufficient coverage causes steric stabilization [7,8,13]. However, a complex and interesting phenomenon occurs in the middle of the coverage range, as explained below.

Aqueous mixed dilute suspensions of inorganic nanoparticles and nonionic polymers exhibit a bistable state, becoming a non-flowable gel-like state when shaken or sheared and returning to a flowable sol-like state with standing, under certain conditions, when the particle surface is not completely saturated with the polymers [14]. Such reversible suspensions are called “shear-induced gels” or “shake-gels” [15]. Shake-gels can be formed with silica nanoparticles [14,16,17,18,19,20,21,22,23,24], montmorillonite/bentonite [18,25], and laponite [15,18,26,27,28] as inorganic nanoparticles. Poly(ethylene oxide) is often used as the polymer to form shake-gels, but nonionic surfactant is also reported to form shake-gels [25]. In any case, the moderate polymer coverage on the particle surface is important for the formation of shake-gels [14]. Phase diagrams of the formation of shake-gels with laponite, montmorillonite, silica nanoparticles, and PEO have been reported [18]. The diagram shows that shake-gel formation occurs only in a narrow range of the amount of polymer dose per surface area of silica (called *C*_p_ in this paper), regardless of particle type, particle concentration, and the molecular weight of the PEO. Consequently, the polymer coverage on the particle surface is suggested to be a dominant parameter for the formation of shake-gels.

In contrast to the formation of shake-gels, some studies have focused on the relaxation process of shake-gels from the gel-like to sol-like state [15,17,21,22,26]. The relaxation time of shake-gels composed of laponite and PEO was measured by direct observation, and the result showed that increasing the PEO concentration decreased the relaxation time [15]. Huang et al. systematically measured the relaxation time of shake-gels composed of silica nanoparticles and PEO using direct observation to elucidate the effect of the amount of polymer dose per surface area of silica *C*_p_ [21]. Their results showed that the relaxation time was longest at *C*_p_ around 0.04 mg/m^2^ irrespective of the PEO molecular weight and pH of the suspensions. The results of the *C*_p_-dependent relaxation time suggest that the value of polymer coverage on particle surfaces is closely related to the relaxation dynamics of shake-gels.

Contrary to the relaxation, the effect of polymer coverage on particle surface on the gelation dynamics has not been fully examined because systematic studies on the relationship between the gelation time and the *C*_p_ of shake-gels are scarce. A shake-gel in the sol-like state composed of silica nanoparticles and PEO showed a sudden increase in viscosity and rapid gelation after a certain time from the commencing of applied steady shear flow, and the reduction in pH shortened this gelation time [19]. Moreover, the gelation time of shake-gels composed of silica nanoparticles and PEO under steady shear flow decreased with increasing shear rate, and the longest gelation time was obtained at a certain range of polymer concentration and temperature [20]. However, these previous studies did not focus on the value of *C*_p_, so the effect of the saturation degree on the gelation time is difficult to consider. Additionally, the experimental conditions were limited to regions of relatively high polymer concentration, and they only considered the gelation dynamics of shake-gels and did not investigate the relaxation. Therefore, the relationship between the gelation and relaxation dynamics was not clear.

In this relation, the way that the flow field is introduced is also an important issue for the gelation of shake-gels. The gelation behaviors in different flow types have been reported. The gelation occurred within a few seconds when the suspension was shaken by a hand or pushed out of a syringe while shearing with a rheometer resulted in a longer time taken for gelation to occur [18]. These reports suggest that the gelation behavior may vary depending on how the flow field is applied. Nevertheless, the relationship between the gelation dynamics and flow types has not been studied in detail.

Based on the lack of previous works described, this study aims to clarify the effect of the value of *C*_p_ on the gelation time of shake-gels composed of silica nanoparticles and PEO. The effects of different flow field types on the gelation of shake-gels, such as handshaking, end-over-end rotation, constant shear flow field, and time-varying shear flow field, are examined [21]. In particular, studying the behavior of shake-gels under well-defined shear flow fields will be useful for the physical understanding of the gelation mechanisms and comparison with numerical simulation in the future [29,30]. The results of gelation time are compared with the results of the relaxation time of previous research [21]. Using *C*_p_ provides a unified index for the dynamics of shake-gels that is not limited to independent particle fractions or polymer concentrations. We believe that these results will provide useful insight into the shear-thickening dynamics of the mixture with silica nanoparticles and PEO.

## 2. Materials and Methods

### 2.1. Materials

LUDOX TM-50 (Sigma-Aldrich, St. Louis, MO, USA, product number 420778) was used as an aqueous suspension of silica nanoparticles without any pretreatment. According to the manufacturer, the silica weight fraction was 50 wt%, and the specific surface area was around 140 m^2^/g. The diameter of silica nanoparticles was reported to be 30–34 nm, and the hydrodynamic diameter was reported to be 32.35 ± 0.22 nm, measured using dynamic light scattering [19,31]. The value of pH was around 9.4, obtained by a compact pH meter (LAQUAtwin pH-22B, HORIBA, Kyoto, Japan). Around this pH, silica particles are negatively charged, and the suspension is charge-stabilized [32,33].

Poly(ethylene oxide) (PEO) is an electrically neutral and linear polymer with molecular formula HO-[CH_2_CH_2_O]_n_-H. In this research, the average molecular weights of used PEOs were 600 kg/mol, 1000 kg/mol, and 4000 kg/mol (Sigma Aldrich, USA, product numbers 182028, 372781, and 189464, respectively). The radius of gyration <*S*^2^>_*z*_^1/2^ (nm) and the overlap concentrations C* (g/cm^3^ %) of PEOs can be estimated from the equations below [28,34]:

(1) $${\langle S^{2}\rangle }_{z^{1/2}}={\left(4.08\times 10^{-4}\times {M_{w}}^{1.16}\right)}^{\frac{1}{2}}$$

(2) $$C^{*}=\frac{3M_{w}}{4\pi N_{A}{\langle S^{2}\rangle }_{z^{3/2}}}$$

where *M_w_* is the molecular weight of the PEO, and *N_A_* is the Avogadro number. The intrinsic viscosity of PEOs can be estimated from the Mark–Houwink equation below:

(3) $$\left[\eta \right]=K{{\times M}_{w}}^{\alpha }$$

where K and α are constants determined by polymer type, solvent type, and temperature. Table 1 shows the radius of gyration <*S*^2^>_z_^1/2^, the overlap concentrations C*, and the intrinsic viscosity $\left[\eta \right]$ for each molecular weight of PEO. The constants for $\left[\eta \right]$ were taken from a report of experiments performed at the same temperatures, $K=0.047745\times 10^{-3}$ cm^3^/g and α=0.662086 [35].

Deionized water was obtained by using pure water production equipment (Elix Advantage5, Millipore, Japan).

### 2.2. Preparation of Shake-Gel Samples

The PEO powder was dissolved in the deionized water at 1 wt% for 72 h under dark conditions using a magnetic stirrer. The PEO solution was used within 3 weeks after the preparation. This operation was performed to take care of the possibility of oxidation and degradation of the PEO molecules.

Shake-gels were prepared by adding the LUDOX TM-50, the deionized water, and the PEO solution in a bottle in that order. Weight fractions of the silica nanoparticle in the mixed suspensions were fixed at 20 wt%. This corresponds to a volume fraction of 10.2 vol%, assuming that the density of silica is 2.2 g/cm^3^. The average surface distance of the nearest silica particles h can be calculated as h≃12 nm, assuming that the particles are distributed in the suspension in a hexagonal close-packed structure [23]. The PEO concentration was changed to set the value of *C*_p_ (the amount of polymer dose per silica surface area) from 0.01~0.15 mg/m^2^. These values correspond to 0.23~3.4 μmol/m^2^ when converted to the number of monomers –[CH_2_CH_2_O]– that make up the PEO. The final weight of the mixture was fixed at 5.0 g to observe the variation in flowability by the end-over-end rotation test and 18 g for viscosity measurements using a viscometer. Mixing was performed using a test tube mixer (PresentMixer, TAITEC, Kyoto, Japan) for the preparation of 5.0 g samples and shaking by hand for the preparation of 18 g samples. In the processes of these preparations, the samples of *C*_p_ above 0.015 mg/m^2^ became gel-like and became elastic enough to hold their own weight a few seconds after shaking by hand. The samples of *C*_p_ = 0.01 mg/m^2^ were sol-like. These gelled samples relaxed to sol-like states by being kept still for over 24 h under dark conditions. The pH values of the mixtures were around 9.5. This pH value is within the range of pH for shake-gel formation reported in previous studies, where silica nanoparticles had certain repulsive forces with each other [19,21].

### 2.3. Observation of Flowability Change by End-over-End Rotation

Capped test tubes containing 5.0 g of the silica–PEO sample in a sol-like state were set vertically to a rotational mixer at first (LABORATORY HIGH POWER MIXER, AS ONE, Japan) as the tube center was located at the height of the axis of rotation and was rotated at 60 rpm. The rotational axis was set horizontally. The diameter of the test tubes was about 1.5 cm, and the height was about 10.5 cm. In the early stages of rotation, the suspension was able to flow from one end to another end of the test tube, but the flowability decreased with time and eventually stopped flowing to the end of the test tube. The time from the start of the rotation to the suspension no longer reached the one end of the test tube was defined as the time until the flowability decreased.

### 2.4. Viscosity Measurements

The suspensions in the sol-like state were applied to shear flow by using a concentric double-cylinder viscometer (Merlin VR, Rheosys, Hamilton Township, NJ, USA). The viscosity was measured as a function of time or shear rates. The suspension was placed in the viscometer and left to settle for 15 min under shading conditions after the temperature had been stable at 20 degrees before the measurement began. This manipulation was performed to offset a history of shear applied to the sample as it was set in the viscometer. The viscosities were obtained every second when the viscosities were measured as a function of time at a constant shear rate. For some samples requiring long measurements time, the viscosities were obtained every 2 s. The viscosities were also measured as a function of the shear rate to obtain flow curves. In this case, the shear rates logarithmically changed from 10 to 2000 s^−1^ in 40 steps, and each measurement was performed for 30 s. The top of the concentric double-cylinder was open to the atmosphere, allowing visual observation of the sample during the measurement.

All the experiments, including the preparation of samples, were performed at a room temperature of 20 °C.

## 3. Results

### 3.1. On Visual Changes of Prepared Suspension

In the processes of preparations, samples of *C*_p_ above 0.015 mg/m^2^ became cloudy and gel-like, which supported their own weight when inverted. On the contrary, samples of *C*_p_ = 0.01 mg/m^2^ did not become gel-like. These results are almost consistent with previous studies that reported the formation of shake-gels with silica nanoparticles (LUDOX TM-50) and PEO [18,19,21,23].

The gel-like samples relaxed to their sol-like states by being kept unmoved for over 24 h under shaded conditions, but some differences were observed in the states after relaxation. The samples of *C*_p_ below 0.03 mg/m^2^ were relaxed but slightly sticky and cloudy states, which made it difficult to read text clearly through the samples, while the samples of *C*_p_ above 0.05 mg/m^2^ were completely relaxed, highly fluid, and translucent enough to read text clearly through the samples. The minor sticky and cloudy conditions were still observed more than a week later.

These observed results suggest that the stable state of the shake-gels after relaxation depends on the value of *C*_p_. In a mixed dispersion of adsorptive polymers and colloidal particles, generally, bridging flocculation occurs at low concentrations of polymers when a polymer adsorbs on multiple particles and forms bridges [9]. In the samples of *C*_p_ below 0.03 mg/m^2^, some aggregate structures might remain stable structures after relaxation from a gel-like to sol-like state, which is thought to cause the minor turbidity and stickiness of the suspension. Huang et al. reported that shake-gels composed of silica nanoparticles and PEO form stable structures with long relaxation times at *C*_p_ around 0.04 mg/m^2^ [21]. Considering these facts together, we suggest that the conditions under which such aggregate structures tend to remain may contribute to the longest relaxation time.

### 3.2. Observation of Flowability Change by End-over-End Rotation

The end-over-end rotation test revealed that some suspensions, which initially flowed down to the bottom end of the test tube, stopped flowing down to the end of the tube and stayed on the side wall of the tube over time. These visual decreases in flowability were observed for the samples of *C*_p_ above 0.015 mg/m^2^ within an hour, but the samples with *C*_p_ = 0.01 mg/m^2^ did not exhibit a decrease in flowability. That is, only the samples that were transformed into the gel-like state by being hand-shaken during preparation decreased in flowability.

**Figure 1:**
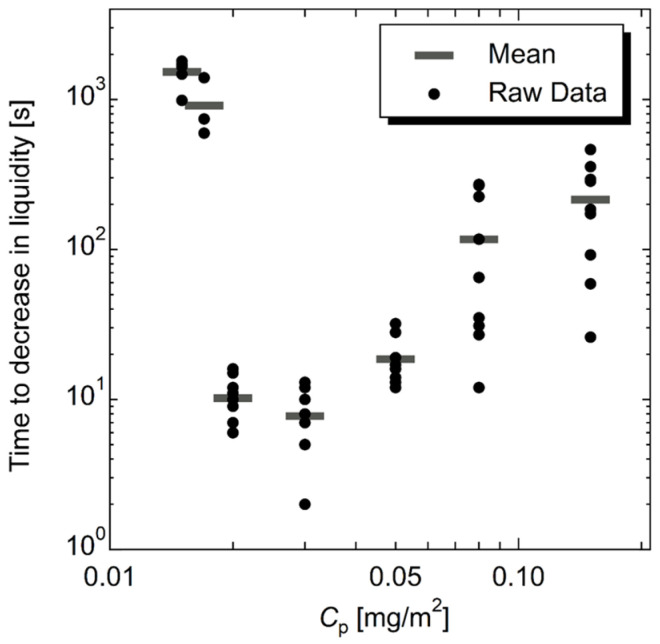
The time to decrease in liquidity was plotted against the value of *C*_p_ (the amount of polymer dose per silica surface area). Horizontal bars represent the mean value. The PEO molecular weight was 1000 kg/mol.

The time to the reduction in liquidity was plotted against the value of *C*_p_, as shown in Figure 1. The time to decrease in liquidity was minimum at *C*_p_ = 0.03 mg/m^2^, and the increase or decrease in the *C*_p_ from 0.03 mg/m^2^ resulted in monotonical increases in the time to the decrease in liquidity. This trend demonstrates that the time to decrease in liquidity, that is, gelling time, of shake-gels reaches a minimum value at a certain *C*_p_. These data are the first reported to indicate the existence of the minimum time to decrease in liquidity of shake-gels at a certain *C*_p_.

Although we could not find any reports applying the end-over-end rotation test to shake-gels, a previous study used the end-over-end rotation test to evaluate the coagulation rate of colloidal particles in the presence of turbulent flow [36,37,38]. To estimate the effective shear rate by the end-over-end rotational flow, they used the rate of energy dissipation per unit mass $\epsilon_{T}$ as $\epsilon_{T}=ghf$, where g is the gravitational acceleration, h is the pouring height, and f is the pouring frequency [39]. The energy dissipation $\epsilon_{T}$ can be used for dimensional analysis to evaluate the local shear rate $G={\left(\epsilon_{T}/\nu \right)}^{1/2}$, where ν is the kinematic viscosity. However, for shake-gels, the effective shear rate cannot simply be estimated from such the theory because their kinematic viscosity is difficult to determine. This is because shake-gels are non-Newtonian fluids, as discussed below. However, assuming Newtonian fluid, we can estimate the effective shear rate. Assuming that $f=2\;s^{-1}$, h=7.5 cm (sample height is 3 cm), and $\nu =10^{-6}\;m^{2}s^{-1}$ as the kinematic viscosity of water at 20 °C, the effective shear rate G can be calculated to be 1212 s^−1^. Since the kinematic viscosity of shake gel is considered higher than that of water at the same temperature, the actual effective shear rate should be less than 1212 s^−1^.

### 3.3. Critical Shear Rate

**Figure 2:**
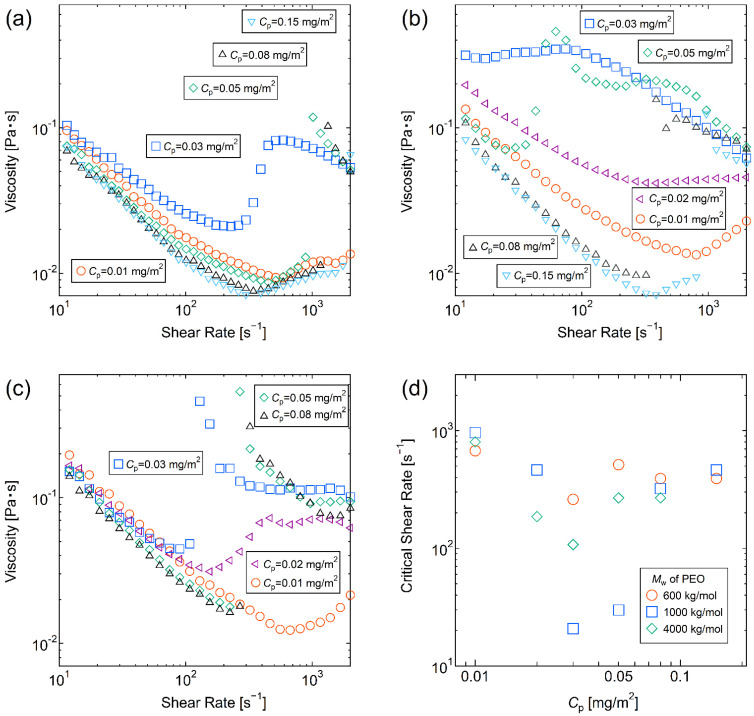
(**a**–**c**) Viscosities were plotted against the applied shear rate at different *C*_p_ values. The molecular weights of PEO were (**a**) 600 kg/mol, (**b**) 1000 kg/mol, and (**c**) 4000 kg/mol, respectively. (**d**) Critical shear rates were plotted against the *C*_p_ values at different PEO molecular weights. The PEO molecular weights were (○) 600 kg/mol (□), 1000 kg/mol, and (△) 4000 kg/mol, respectively.

Figure 2a–c shows the viscosities of the silica–PEO suspension plotted against the applied shear rate at different *C*_p_ and the PEO molecular weights, 600 kg/mol, 1000 kg/mol, and 4000 kg/mol, respectively. In the low shear rate region, the samples exhibited shear thinning, in which the viscosity decreased with increasing the shear rate. A further increase in shear rate induced shear thickening, where the viscosity increased with increasing shear rate. In particular, the samples with *C*_p_ above 0.05 mg/m^2^ showed discontinuous and abrupt shear thickening regardless of the PEO molecular weight, and the samples with *C*_p_ = 0.03 mg/m^2^ also showed discontinuous and abrupt shear thickening for the PEO molecular weight of 600 kg/mol and 4000 kg/mol. Critical shear rate is defined as the shear rate at which the shear viscosity turns from shear thinning to shear thickening. The critical shear rates are plotted against *C*_p_ in Figure 2d. The critical shear rates were minimum at *C*_p_ = 0.03 mg/m^2^ independent of PEO molecular weight.

At the same time, with the discontinuous and abrupt shear thickening, the samples visually changed from sol-like to gel-like states. Specifically, the translucent suspensions became cloudy and viscoelastic, rising to wrap around an axis of rotation of the inner cylinder of the viscometer. The rising phenomenon was more pronounced with the larger molecular weights of PEOs and the higher *C*_p_ values. The rising effect is called the Weissenberg effect or the rod-climbing effect, and this phenomenon is considered to be caused by the elongation of the polymers in the shear flow, which generates normal stress differences toward the inner cylinder [40,41]. Thus, the observation of the Weissenberg effect could be indirect evidence that the shear flow field generated by the viscometer caused the PEO elongation. The cloudiness of the suspension is thought to be due to the formation of some larger structures in the suspension, which scatter the light more strongly.

### 3.4. Gelation Time by Applying Constant Shear Flow

**Figure 3:**
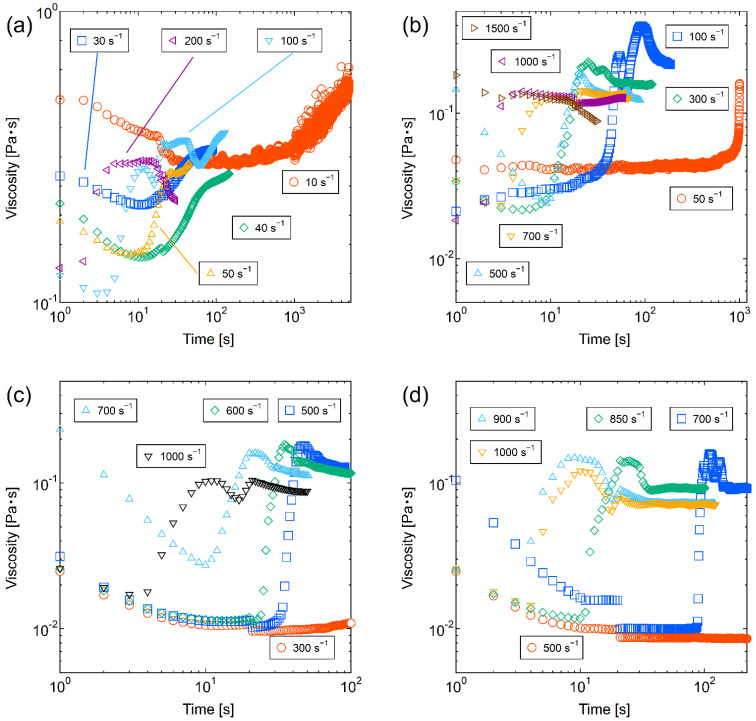
Temporal changes in viscosity of shake-gels under steady shear rates. The *C*_p_ values (the amount of polymer dose per silica surface area) were (**a**) 0.03 mg/m^2^, (**b**) 0.05 mg/m^2^, (**c**) 0.08 mg/m^2^, and (**d**) 0.15 mg/m^2^, respectively. The PEO’s molecular weight was 1000 kg/mol.

Figure 3 shows temporal changes in the viscosity of the silica–PEO suspension subjected to a steady shear rate by the viscometer. The values of *C*_p_ were (a) 0.03 mg/m^2^, (b) 0.05 mg/m^2^, (c) 0.08 mg/m^2^, and (d) 0.15 mg/m^2^, respectively. The PEO’s molecular weight was unified at 1000 kg/mol. The shear rates were below the critical shear rates estimated from the flow curves. Almost all the samples initially exhibited thixotropy, in which the viscosity decreased with time. As additional information, we should mention that the discontinuous decrease in viscosity around 20 s after the start of shearing was most likely a characteristic of the viscometer we used. At certain points, the samples exhibit rheopexy, in which the viscosity increases with time.

**Figure 4:**
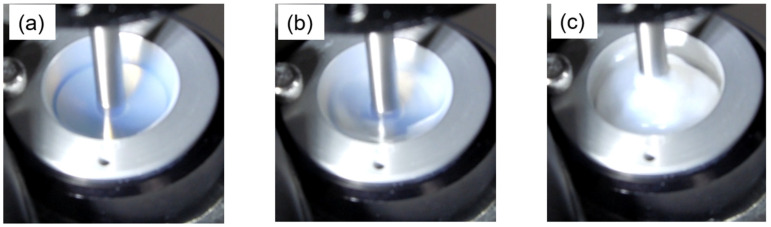
Time series photographs of shake-gels under applied shear by the viscometer. The molecular weight of PEO was 1000 kg/mol, and the value of *C*_p_ was 0.05 mg/m^2^. The constant shear rate of 300 s^−1^ was applied, shown as the symbol (◇) in Figure 3b. (**a**) At the beginning of shear (1–16 s), viscosity was decreasing. The suspension was transparent. (**b**) At medium-term shear (17–30 s), viscosity turned from decreasing to increasing. Some white gel-like segments were observed. (**c**) At the end-term of shear (after 30 s). Viscosity increased and reached a constant. Whole gelation and the Weissenberg effect were observed.

Some of the samples showed gradual rheopexy before showing rapid rheopexy, as shown in Figure 3b, around the shear rates of 50–100 s^−1^. In contrast, most of the observed rheopexy was rapid, and in some cases, the viscosity increased nearly 10 times in a few seconds. That is, so-called shear-induced gels or shake-gels formed. The smaller the shear rate is, the longer the time required for the rheopexy is. These trends agree with previous studies focusing on the gelation time of shake-gels [20,25]. Visual changes in the suspensions were also observed, as seen when the flow curves were obtained. Thus, with the increasing viscosity, the sol-like suspension changed to a gel-like state, and the Weissenberg effect was also observed, as shown in Figure 4. However, there is a slight difference from when the flow curve was obtained. In the measurement of flow curves, the visual changes occurred almost simultaneously with the increase in viscosity.

Despite this, in the measurement under steady shear, no visual changes occurred in the region of slowly increasing viscosity, while the visual changes occurred in the region of rapidly increasing viscosity. This trend was more pronounced for the lower shear rates. In consequence, we consider that the rapid increase in viscosity appeared at the time when the entire suspensions took on gel-like states. After rheopexy occurred, some samples showed oscillating viscosity values. In the region, the viscosity oscillations occurred probably because of the instability of flow called rheochaos or the slippage between the gel-like sample and the inner wall of the viscometer [42,43].

**Figure 5:**
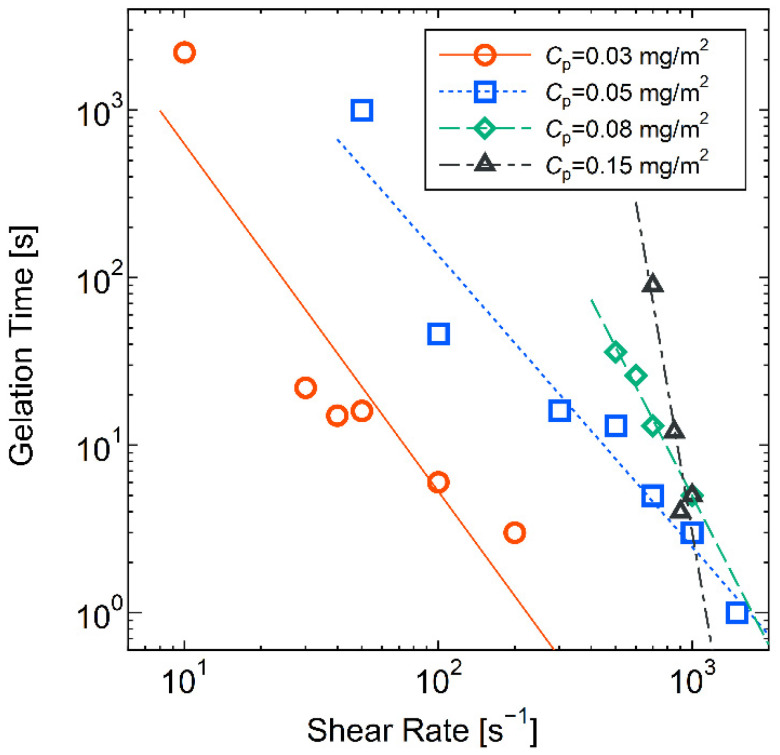
Relationships between applied shear rate and the gelation time at which temporal increase in viscosity value was maximum at different *C*_p_ with (○) 0.03 mg/m^2^, (□) 0.05 mg/m^2^, (◇) 0.08 mg/m^2^, and (△) 0.15 mg/m^2^, respectively. Each plot was obtained from the experimental data in Figure 3, and the dotted lines were approximated using the least-squares method. The PEO molecular weight was 1000 kg/mol. The samples with *C*_p_ values smaller than 0.03 mg/m^2^ did not change to a gel-like state under the steady shear.

Focusing on the point at which the viscosity increased most rapidly, the results from Figure 3 are summarized in Figure 5 as gelation time versus the applied shear rate. The gelation time was defined as the point at which the rate of increase in viscosity was largest near the rapid rheopexy because the visual gelation of the suspension occurred when the increase in viscosity was rapid. There are power–law relationships between the gelation time and shear rate approximated in Equation (4):

(4) $$t_{G}=k{\overset{˙}{\gamma }}^{a}$$

where $t_{G}$ corresponds to the gelation time and $\overset{˙}{\gamma }$ to the shear rate, the values of k and a are constants, and the a corresponds to the slopes in the double logarithm graph. The parameters determined using the least-squares method are summarized in Table 2 for each *C*_p_. Note that the $t_{G}$ and $\overset{˙}{\gamma }$ are dimensionless numbers that are pre-divided in their dimensions. Therefore, the constants k and a are also dimensionless.

In the range of *C*_p_ = 0.05~0.15 mg/m^2^, the magnitude of the slope a increases with increasing *C*_p_, meaning that the gelation time sharply increases with decreasing shear rate for higher *C*_p_. For the sample of *C*_p_ = 0.03 mg/m^2^, the value of the slope is not significantly different from that for *C*_p_ = 0.05 mg/m^2^, but the coefficient value k becomes smaller, meaning that the gelation time tends to be the shortest. For samples with a *C*_p_ smaller than 0.03 mg/m^2^, no increase in viscosity was observed under the steady shear using the viscometer. Consequently, the sample with *C*_p_ = 0.03 mg/m^2^ tended to have the shortest gelation time.

A previous study reported that the slopes of both logarithmic graphs of gelation time and applied shear rate for bentonite/surfactant shake-gels are constant for constant mass ratios of the components [25]. Their results partially support that the slope value a is determined by the *C*_p_ value.

## 4. Discussion

### 4.1. Effect of the Value of C_p_

From all the results of experiments, we confirmed that the samples with *C*_p_ = 0.03 mg/m^2^ tend to have the shortest time to decrease in liquidity in the end-over-end rotation test, the lowest critical shear rates for shear thickening, and the shortest gelation time under steady shear rates. In other words, regardless of the applied flow type, the conditions with *C*_p_ = 0.03 mg/m^2^ are the best conditions for the formation of a gel-like state for the shake-gels composed of silica nanoparticles and PEO in the range of *C*_p_ = 0.01~0.15 mg/m^2^. These results suggest the existence of an optimum value of *C*_p_, at which the bridging of silica particles using PEO molecules becomes more pronounced, and the shake-gels are more likely to form.

Huang et al. reported the effect of *C*_p_ on the relaxation time of the shake-gels composed of silica nanoparticles and PEO in the range of *C*_p_ = 0.01~0.15 mg/m^2^ [21]. The results of Huang et al. allowed us to easily compare our experimental results of the gelation time with their results of the relaxation time because the shake-gel samples used in their experiments were exactly the same as those used in this report. They measured the relaxation time of shake-gels from gel-like to sol-like state using direct observation and reported that the relaxation time was longest at *C*_p_ around 0.04 mg/m^2^, irrespective of the PEO’s molecular weight and pH of suspensions. Thus, the conditions for the minimum gelation time and the maximum relaxation time are nearly identical. This agreement suggests that the value of *C*_p_ is a decisive parameter in both the gelation and the relaxation dynamics of shake-gels.

The *C*_p_-dependent relaxation mechanisms were discussed by Huang et al. as follows. At low *C*_p_ values, increasing the dose of PEO promotes network formation by using silica nanoparticles as cross-linkers, which increases the stability of the gel-like state and the length of relaxation time. A further increase in PEO dose, represented as high *C*_p_, reduces available sites for PEO adsorption on silica surfaces and reduces the possibility of the PEO network using cross-linked silica nanoparticles. Thus, the high *C*_p_ values lead to easier desorption of PEO from the surfaces of silica particles and result in decreases in gel stability and a shorter relaxation time.

The gelation of shake-gels is considered to be due to the formation of network structures of PEO molecules throughout the suspension via the elongation of PEO molecules using applied shear flow and the recombining and formation of bridged PEO molecules and silica particles adsorbed on multiple other particles [14,20,29]. That is, applying shear flow to a colloidal suspension containing adsorbable polymers could result in the development of network structures of particles and polymers via bridging, where some adsorbed polymer segments are detached from a particle and replaced by segments of other polymers adsorbing neighboring particles. It is also mentioned that the surfaces of the particles should be unsaturated by the polymer for shear-induced gelation [14,18].

The effect of *C*_p_ values on the ease of gelation can be discussed as follows. Smaller *C*_p_ values result in a lower coverage of PEO molecules on silica surfaces and prevent gelation since complex network formation is less likely to occur. *C*_p_ values that are too high result in higher coverage of PEO on silica surfaces and prevent the gelation since succeeding adsorption of PEO molecules on silica surfaces is less likely to occur. The larger or longer shear flows are necessary to promote collision of the contents or to stretch the PEO sufficiently. Conversely, the moderate *C*_p_ values mean that adsorbable sites remain on the silica particle surface, and concurrently sufficient PEO molecules exist to form the gelation network. In this moderate condition, the recombination of PEO molecules is more likely to occur, and gelation is more likely to occur even under weak shear or a short time of shear.

There may be room for further improvement to the method of judging whether the samples are in sol or gel states. In this study, visual observation, shear thickening, and rheopexy were used as the criteria for gelation. Since the results were not based on viscoelasticity, it is debatable whether they really judge gels or sols. For this reason, we used the notation “gel-like” and “sol-like”. However, even without evaluation of viscoelasticity, the shear thickening and rheopexy were a phenomenon that does not normally occur in such dilute suspensions. Therefore, we believe that it is also significant enough to focus on the increase in viscosity. As more advanced research, the determination of gels and sols using viscoelasticity is also expected in the future [17,18,26].

### 4.2. Effect of the Different Flow Types

Samples with a *C*_p_ above 0.015 mg/m^2^ easily changed to gel-like states within 3 s when shaken using the test tube mixer and within 10 s when shaken by hand. In the case of end-over-end rotation, the samples changed to gel-like states from a few seconds to more than 1000 s at the longest. Moreover, in the case of the constant shear flow in the viscometer, the increase in viscosity occurred from a few seconds to more than 1000 s. Moreover, the samples with *C*_p_ below 0.02 mg/m^2^ did not show a rise in viscosity with time, while the gradual shear thickenings were observed even at low *C*_p_ when the flow curves were obtained under varying shear rates. It has been noted in several previous studies that the gelation behavior of shake-gels differs depending on the type of applied flow fields. In fact, some previous studies reported that gelation of shake-gels did not occur under simple Couette flow in a rheometer, while the gelation easily occurred using manual agitation at an estimated shear rate of less than 100 s^−1^ or when pushed out from a syringe [18,26].

These results suggest that the variation of flow fields applied to the suspension has a significant effect on shake-gel formation and the time to gelation. On the one hand, the shear flow fields applied by the viscometer were well-defined Couette flow fields. On the other hand, the mixings using a test tube mixer, shaking by hand, and end-over-end rotation were all considered turbulent flows where the suspension flow fields change in time and space. This consideration suggests that vigorous turbulent flow fields are more effective in inducing gelation than steady shear flow fields.

The reasons why the transitions to gel-like states were difficult under steady shear flow are considered as follows. The shear flow field causes the elongation of PEO molecules, and silica nanoparticle–PEO molecule collisions give rise to the formation of small flocs or aggregates. The flocs were expected to be aligned along the shear flow [24]. This orientation might reduce collisions between the flocs and prevent the flocs from growing into larger network formations [26]. The slight increases in viscosity before the rapid increases suggest that the floc growth’s network formation proceeds very slowly [30]. The rapid increase in viscosity and changes to the gel-like state occur when the floc formation progresses to some extent, and finally, shear force can exceed a threshold energy barrier [30]. The threshold value is proposed as “gelation energy” that must be reached before gelation occurs, and that is responsible for the long gelation times observed at low shear rates [20].

Under turbulent flow conditions, flow velocity and pressure fluctuated irregularly in both time and space. Hence, large local shear stress is exerted on the PEO molecules or the silica–PEO flocs. Therefore, the turbulent flow facilitates the gelation of shake-gels as the PEO becomes more elongated and components collide with each other more frequently. While some samples did not exhibit rheopexy under the steady shear, the samples of the same component showed shear thickening in the flow curves. This opposed result suggests that changing the shear rate during the measurement of the flow curve is expected to disrupt the instantaneous flow field and promote the silica–PEO structure’s collision and growth.

Even in the steady shear experiments, we should mention that turbulence could occur due to the following factors. The double–cylinder viscometer used in this study employs a drive system in which the inner cylinder rotates to generate shear flow. It is known that double cylinders with a rotating inner cylinder are less likely to generate laminar shear flow than those with an outer rotating cylinder [44]. It has also been noted that, even with high viscosity and a low Reynolds number, the elasticity of polymers can lead to unstable flow [44]. The elastic forces generated by the elongation of the PEO molecules in the flow field may destabilize the Couette flow in the viscometer, which in turn may facilitate collisions between the flocs.

## 5. Conclusions

We investigated the gelation dynamics of the shake-gels composed of silica nanoparticles and poly(ethylene oxide) (PEO) with focusing on the effect of *C*_p_, the amount of polymer dose per surface area of particles. The observation of the flowability changes in the end-over-end rotation test revealed that the gelation time was the shortest at *C*_p_ = 0.03 mg/m^2^ in the range of *C*_p_ = 0.01~0.15 mg/m^2^ for the first time. Similarly, the gelation time under steady shear in a viscometer tended to shorten as *C*_p_ approached 0.03 mg/m^2^. Furthermore, the critical shear rate was minimized at *C*_p_ = 0.03 mg/m^2^, irrespective of the molecular weight of the PEO. To summarize, the gelation behavior depended on the *C*_p_ values, and we found that the gelation time and the critical shear rate were the minima at *C*_p_ = 0.03 mg/m^2^. These results demonstrate that the amount of PEO dose per surface area of silica is crucially important to control the mechanism of shake-gel formation. The present study also suggests that the behavior and time taken for gelation differ depending on the flow field presented to shake-gels in sol-like states. In particular, the difference between well-defined flow fields and turbulences may be important.

## Figures and Tables

**Table 1:** The radius of gyration <*S*^2^>_*z*_^1/2^ and the overlap concentrations C* for each molecular weight of PEO.

*M_w_* (kg/mol)	<*S*^2^>_*z*_^1/2^ (nm)	C* (10^−3^ g/cm^3^)	[η] (cm^3^/g)
600	45	26	0.32
1000	61	17	0.45
4000	136	6.3	1.12

**Table 2:** The parameters of the power-law approximation are calculated using Equation (3), where $t_{G}$ corresponds to the gelation time and $\overset{˙}{\gamma }$ to the shear rate. The values of *k* and *a* are the constants of the power–law relationship, and the values of *a* correspond to the slope of the graph. The values of R^2^ are the coefficient of determination for the least-squares method.

*C*_p_ (mg/m^2^)	*k* (-)	*a* (-)	R^2^
0.03	7 × 10^4^	−2.07	0.993
0.05	4 × 10^5^	−1.74	0.938
0.08	3 × 10^9^	−2.95	0.967
0.15	1 × 10^27^	−8.88	0.995

## Data Availability

The data are available from the authors upon reasonable request.

## Sample Availability

Materials are commercially available from the manufactures.
